# Competency lists for urban general practitioners/family physicians using the modified Delphi method

**DOI:** 10.1186/s12875-023-01984-z

**Published:** 2023-01-19

**Authors:** Toshichika Mitsuyama, Daisuke Son, Masato Eto, Makoto Kikukawa

**Affiliations:** 1 Department of Medical Education Studies, International Research Center for Medical Education, Graduate School of Medicine, The University of Tokyo, 7-3-1 Hongo, Bunkyo-ku, Tokyo, 113-0033 Japan; 2 Department of Community-based Family Medicine, Faculty of Medicine, Tottori University, Yonago, 683-8503 Japan; 3 Department of Medical Education, Faculty of Medical Sciences, Kyushu University, 3-1-1 Maidashi Higashi-ku, Fukuoka, 812-8582 Japan

**Keywords:** General practitioner, Family physician, Competency, Urban area, Non-urban/rural area, Delphi method

## Abstract

**Background:**

In recent years, the growing global urbanization and urban population have resulted in the emergence of various health problems unique to urban areas. Therefore, training general practitioners and family physicians who can tackle the complex health problems of urban areas and improve the health of urban people is one of the most important issues of our time. However, findings on competencies for urban general practitioners (GP) and family physicians (FP) were limited. This study aimed to identify their comprehensive and content-validated list of competencies.

**Methods:**

We used the modified Delphi method to develop a content-validated competency list. First, we analyzed and synthesized the competencies extracted from the literature review using qualitative thematic analysis methods to create an initial competency list of 34 items. We then assembled 39 expert panelists in four groups of study participants: physicians, nurses, patients, and medical education specialists. The expert panelists were asked to indicate their level of agreement with the lists and provide revised comments on the description of each competency via a web-based questionnaire. Their responses were analyzed quantitatively and qualitatively by the research team and used to revise the list. These processes were repeated, and the survey was completed when it was determined that consensus had been reached.

**Results:**

Three rounds of Delphi were conducted. 39 responded in the first round, 38 in the second round, and 36 in the third round. The initial list of competencies was revised and consolidated from 34 to 14 items in the first round, bringing the total to 20 items along with six new items proposed by the panelists. In the second round, it was revised and consolidated into a list of 18 items. In the third round, all 18 items were considered to have been agreed upon by the panelists, so the survey was closed.

**Conclusion:**

We identified a comprehensive 18-item list of competencies for urban GP/FP in a content-validated manner. Several are newly discovered competencies in this study. The findings of this study will be useful for the future training of urban GP/FP and for solving urban health problems.

**Supplementary Information:**

The online version contains supplementary material available at 10.1186/s12875-023-01984-z.

## Background

In general, general practitioners and family physicians (GP/FP) competencies reflect the characteristics and social needs of the health-responsible population. However, the population characteristics are changing. In particular, a major social change in recent years is the global urbanization and concentration of population in cities. As of 2018, 55% of the world’s population, or 4.2 billion people, live in urban areas, and by 2050, the urban population is projected to grow to 68%, or 6.7 billion people, due to the increasing urbanization of countries’ settlements and the growth of the world’s population [1, 2]. Moreover, various health problems specific to urban areas have been pointed out. For example, the risk of some diseases due to urban living conditions such as lack of housing and exercise space, sanitation problems, and air pollution, as well as the lack of adequate health care services due to socioeconomic reasons associated with increasing disparities in education and economic levels [3]. In addition, the aging of the urban population is also an issue in developed countries, and care provision for the elderly is an important theme in urban primary care. For example, in OECD countries, 56% of the elderly over 65 years old lived in urban areas as of 2010; the percentage continues to increase [4].

Therefore, it is necessary to increase the number of community-responsive and culturally competent GP/FP to improve access to primary care for urban residents [3]. Clarifying the competencies of urban GP/FP that can contribute to improving the health and quality of life of urban people is an important issue in both family medicine and medical education.

Competency is a set of practical abilities enabling professionals to respond to certain social needs. Competency-based medical education (CBME) is a method of educational design that focuses on ensuring that learners have these competencies at the end of the curriculum [5]. Competency is one of the most important pedagogical concepts in training primary care physicians, including GP/FP. Various knowledge about GP/FP competencies has been accumulated so far.

However, previous studies on the competencies of urban primary care physicians are limited. Some of the available literature is limited to examining partial clinical aspects of urban primary care physicians. Few studies have attempted to comprehensively identify the competencies of urban GP/FP from a pedagogical perspective [6]. In addition to research articles, other references include the outline of an urban GP/FP educational program titled “Urban/Inner-City Training Program in Family Medicine” published on the American Academy of Family Physicians (AAFP) website [7]. The guidelines call for training to provide care to the urban underserved and provide culturally effective community-responsive primary care. However, they have a limitation. They are recommendations for designing training curricula and not comprehensive competencies examined by educational research.

Therefore, to train the quality-guaranteed urban GP/FP, a worldwide requirement in the future, pedagogical research clarifying a comprehensive list of competencies in a way that ensures a certain level of content validity is necessary. We reviewed the existing literature and gathered opinions from experts using the modified Delphi to create a comprehensive and content-validated competency list for urban GP/FP.

Since the data for this study was collected in Japan, a brief description of the characteristics of urbanization and health care in Japan is provided below to facilitate adaptation in other countries. Japan has some of the most densely populated cities in the world and is the most aging country in the world [4, 8]. One of the features of the Japanese healthcare system is that most citizens can receive relatively inexpensive, high-quality medical care through the universal health insurance system. Another feature is that patients can freely choose their medical institutions because there is no strict gatekeeper system [9]. Until recently, primary care in Japan has been provided mainly by physicians specializing in other fields, such as internal medicine and pediatrics, who moved from hospitals to clinics as a second career [10]. The bord-certification was established in 2009 by the Japanese Association for Primary Care Alliance to create a quality-assured certification and education system for primary care. Newly trained physicians are now registered as family physicians, and some existing physicians certified as competent to educate family medicine residents are registered as supervising physicians. In addition, in 2018, a general practitioner was added as a new 19th major clinical discipline in a new bord-certification system developed at the initiative of the government.

## Methods

### The modified Delphi method

This study adopts the modified Delphi method, one of the consensus methods. The Delphi method gathers a group of experts on a problem and systematically obtains consensus-based opinions using questionnaires [11–13]. Its characteristics are that the participants (called panelists) answer the questionnaire in several iterations (called rounds), the panelists remain anonymous, and the panelists receive feedback on their overall answers in each round and have the opportunity to revise their answers. While adhering to the above basic principles, there are various variants of the Delphi method to suit research purposes, which are collectively referred to as the “modified Delphi method” [13, 14].

In particular, this study employed a modified Delphi method to create a list of competencies for urban GPs/FPs, which differs from the classical Delphi method in the following two features. First, due to insufficient existing knowledge on the topic, our research team conducted a literature review in advance and prepared a draft of the competency list [15]. Second, the emphasis was on refining the description of the list by analyzing the qualitative revised opinions of the panelists along with the quantitative analysis of the degree of agreement on each competency list presented by the panelists using the Lickert scale [11, 15]. The development and implementation of these research plans were performed with the advice of experts in the modified Delphi method. This study was conducted between March 23 and September 6, 2019.

### Initial list development through literature review

To begin with, our research team conducted a literature review on “competencies specifically needed by urban GP/FP” following a general methodology and extracted competencies from the literature corresponding to the theme [16]. For the literature review, three main categories were defined: “Urban,” “Competence,” and “Primary Care or General Physicians or Family Physicians”; related terms were also listed. Literature searches were limited to [Title/Abstract] and [MeSH Terms] using PubMed. In addition, we added the literature for the three individual competencies: “Underserved,” “Integration of care,” and “Cultural Competence.” These three were chosen because they were competencies derived as representatives in our preliminary study [17]. Inclusion criteria were that the primary research purpose of the searched article was urban GP/FP competency and the article was in English; exclusion criteria were that the research setting was not urban, not primary care, not GP/FP/Nurse Practitioner, and the research topic was not medical education. In addition, we excluded cases where the full text was not available and was not an English paper. The search formula is shown in Additional File 1.

Subsequently, using the thematic analysis method, one of the methods for analyzing qualitative data, descriptions corresponding to the competencies of urban GP/FP were coded from the text of each document, and themes were defined by linking multiple codes together. After translating the extracted themes related to the competencies into Japanese, they were classified and integrated to create an initial competency list. These initial lists were created through discussions among multiple research members [11, 18].

### Participants

The sampling of panelists in the Delphi method should focus on their quality as panelists rather than on quantitative homogeneity by statistical methods. The quality of panelists is defined as the importance of having a heterogeneous group of people relevant to the research topic with diverse attributes and different opinions. In clinical research, clinicians, researchers, and patients are often considered “expert panelists” [19].

In this study, we selected four groups through purposive sampling: GPs/FPs and nurses working in urban areas, medical education specialists, and patient representatives. The overall number of participants was targeted to be at least 30, following previous medical education studies. In particular, we aimed to secure a total of at least 20 GPs/FPs from various urban practice areas. In particular, we adopted the most stringent definition of an urban area as defined by the Statistics Bureau of the Ministry of Internal Affairs and Communications in Japan: a “central city” of a “metropolitan area” and a “municipality with a population density of 4,000 people/km2 or more” of a “Densely Inhabited Districts” [20, 21].

As a more specific requirement for selection, for urban GPs/FPs, the selection was based on a balance of practice region, years of experience, and gender ratio, in addition to having a certified primary care specialty, working in a defined urban area, and extensive experience in urban practice. In addition, GP/FP defined in this study were Japan Primary Care Association certified family physicians who had completed specialist training in primary care and supervising physicians who were certified as competent to practice primary care and to educate family medicine resident. Since the certification system for family medicine physicians began in Japan in 2009, there are few veteran physicians on the expert list. Therefore, we attempted to incorporate the opinions of physicians with a wide range of experience by adding veteran certified family physicians as panelists in addition to JPCA certified family physicians, who are mostly young. For nurses, those with urban practice experience were selected based on referrals from GPs/FPs of study participants. In addition, we recruited graduate nursing students with experience working in urban clinics and hospitals and academic backgrounds through the co-researchers to ensure that the panelists had experience in diverse settings as nurses. For patient representatives, those who belonged to a representative patient organization in the urban area were asked to participate through the co-researcher. For medical education specialists, we selected those with experience in primary care and familiarity with primary care education, not limited to urban areas.

As a result, 39 panelists and four groups of stakeholders (26 GPs/FPs, five nurses, five medical education specialists, and three patient representatives) were collected through purposive sampling.

Table 1 shows the profiles of the panelists. The actual sampling process for each group of participants is described as follows. For GPs/FPs, we used a list of 527 family medicine specialists and 2511 supervising physicians certified by JPCA published on the society’s website as of April 15, 2019 [22, 23]. The list included the name, the area of work (prefecture), and the name of the hospital where they worked. From the list, 129 family medicine specialists working in urban areas were selected, and 28 family medicine specialists were selected according to the regional and gender ratios in the list. To compensate for the lack of experienced physicians on the list of family medicine specialists, we selected 673 supervisors working in urban areas from the list, and 10 were selected based on a balance of location and years of experience. A total of 38 of these physicians were contacted, and finally 17 family medicine specialists and 9 supervising physicians agreed to participate (26 urban GPs/FPs in total). For the recruitment of nurses with experience practicing in an urban primary care setting, we first obtained three participants through referrals from the GPs/FPs of the study participants. In addition, through a co-researcher, we obtained two graduate nursing students with work experience in urban clinics and hospitals and academic backgrounds to participate in the study. With the advice of the co-researcher, who is familiar with Japanese patient organizations, we also approached two representative organizations to recruit patient representatives and obtained three participants as patient representatives. As for the medical education experts, there were 135 medical education specialists certified by the Japanese Society for Medical Education as of April 2019, but only a few met our requirements, so we contacted them in turn and finished the selection process when we obtained participation from five of them [24].

**Table 1:** Profiles of the panelists

Category	Subcategory	GP/FP	Nurse	Patient	Medical education specialist
Number		26	5	3	5
Gender	male	20	0	2	3
	female	6	5	1	2
Age average		42.3	40.6	47.3	45.4
(min-max)		(32–62)	(30–49)	(40–53)	(36–57)
PGY average		17.4	17.8	23.3	20.6
(min-max)		(9–36)	(8–31)	(18–27)	(13–32)
Clinical setting	clinic	20	2	0	0
	community hospital	4	0	0	2
	university hospital/university	2	1	0	3
	others	0	2	3	0
Metropolitan areas	Sapporo	3	0	0	0
	Kanto	15	5	2	1
	Chukyo	2	0	0	0
	Kinki	4	0	1	1
	Hiroshima	1	0	0	0
	Kitakyusyu・Fukuoka	1	0	0	0
Non-urban areas	Kumamoto	1	0	0	0
	Others	–	–	–	3

### Data collection

We asked each selected participant to evaluate the competency list using a web-based survey. We used SurveyMonkey® as the web-based survey instrument. Panelists had to rate on a 5-point Likert scale the degree to which they agreed that the compiled competency lists were “especially necessary for urban general practitioners and family physicians.” The rating was defined as “5” being totally agreeable, “3” neither agree nor disagree, and “1” totally disagree. We also asked the panelists to provide an open-ended commentary on the definition and descriptions for each competency list. For the first round only, we also asked the panelists to suggest up to five new competencies they considered important that were not included in the initial list.

During the entire survey process, only the researcher was aware of the study participants and could view each participant’s responses. Participants did not know the names of other participants or the contents of their responses. They could not view the results of other participants’ responses or the summary of the results sent by the researcher for each round and responded to the next round independently of the other participants.

### Data analysis

Our research team anonymized the collected responses by separating the respondent’s name from the responses in each round and then conducted two main levels of analysis. First, the list was excluded or ranked based on the quantitative data of the mean and standard deviation on the assessment of the degree of agreement by the Likert scale. We decided to exclude those with a mean value of 3.5 or less or standard deviation > 1 on the Likert scale for each competency list in this study, referring to previous studies using the Delphi method in the field of medical education [25]. Second, the qualitative data on the panelists’ revised opinions on each competency list and suggestions for new lists were qualitatively analyzed using the thematic analysis method; the lists were revised and integrated to create a revised proposal for the competency lists [11, 18]. The qualitative analysis results were reviewed among the research members at each round.

At the beginning of each round, we sent a summary of the results of the previous round’s analysis and the revised draft of the new list to the panelists and asked them to evaluate the new list again. The process of iterative revision of the list by the researcher and the panelists was repeated, and the survey was terminated when no exclusion items appeared on the developed competency list, and the panelists were considered to have reached a consensus.

## Results

### The initial list by literature review

We conducted a literature review to examine 629 articles and obtained 53 references according to the inclusion and exclusion criteria between March 23 and April 20, 2019. Our research team used thematic analysis to extract competency statements from these articles, categorized and integrated them by theme, and created an initial list of 34 competencies translated into Japanese Table 2. Based on these initial lists of 34 items, we started the Delphi round.

**Table 2:** Initial list

Round1 -No.	Definition of competency “Urban GPs/FPs”
R1–1.	can provide care that takes into consideration various cultural backgrounds.
R1–2.	can understand various occupations and lifestyles in the city.
R1–3.	can form an appropriate consensus with patients with diverse values ​​in urban areas.
R1–4.	can understand the social context of Urban Underserved Communities.
R1–5.	can provide Urban Underserved Communities with comprehensive, integrated care through multidisciplinary collaboration.
R1–6.	can provide preventive care for Urban Underserved with integrated complementary and alternative medicine.
R1–7.	can communicate effectively with non-living families.
R1–8.	can flexibly provide comprehensive care according to the needs of the patient and the situation of the surrounding medical institution.
R1–9.	can make appropriate hospital referral decisions according to the patient’s situation.
R1–10.	can take responsibility for integrated management for patients who have a division of care due to consultations with multiple specialized departments.
R1–11.	can provide integrated care by primary care for the division of care in the elderly who visit multiple specialized departments.
R1–12.	can work with multidisciplinary and community care resources in mental health to build integrated care teams based on the Patient-centered medical home.
R1–13.	can adequately transition care from pediatrics to young people with disabilities.
R1–14.	can provide HIV patients with integrated primary and mental health care.
R1–15.	can keep track of local social services and their providers for patients.
R1–16.	can collaborate with a wide variety of medical, long-term, and welfare personnel.
R1–17.	can identify community issues that are characteristic of the city and implement a community-oriented approach.
R1–18.	can tackle the challenges of regional alliances in urban emergency care.
R1–19.	can diagnose and treat occupational health problems.
R1–20.	can provide an appropriate initial response to patients with suspected tuberculosis.
R1–21.	can work with health centers to adequately treat outpatient tuberculosis.
R1–22.	can screen for risk factors associated with HIV infection.
R1–23.	can consult with HIV patients in consideration of their culture and values.
R1–24.	can adequately assess and address the risk of suicide in young people.
R1–25.	can screen children for mental health (depression, developmental disabilities, etc.)
R1–26.	can effectively collaborate with multiple occupations on pediatric mental health (depression, developmental disabilities, etc.).
R1–27.	can provide appropriate assessments and referrals to psychiatry regarding mental health issues in the elderly.
R1–28.	can provide psychotherapeutic interventions for culturally diverse older people.
R1–29.	can provide adequate mental health care to racial minority groups.
R1–30.	can make a systematic assessment of mental problems, including refugee PTSD.
R1–31.	can properly diagnose and manage dementia.
R1–32.	can improve the quality of life of patients with dementia.
R1–33.	can provide guidance to obese patients using behavioral transformation theory.
R1–34.	can properly diagnose and treat traffic injuries.

### Results of the modified Delphi method

Consequently, three rounds were conducted between April 26 and September 6, 2019. Initially, 39 panelists participated, 39 responded in Round 1, 38 in Round 2, and 36 in Round 3. Additional file 2 shows the descriptive statistics data and list editing process for each round in the modified Delphi method. An initial list of 34 items was consolidated into 14 items in the first round, which, together with the six newly proposed items, resulted in a list of 20 competencies required for urban GP/FP. In the second round, the list was merged into an 18-item list. In Round 3, all 18 items were considered to have been agreed upon by the participants, and the study was closed. We obtained a list of 18 items with high validity as competencies specifically needed for urban GP/FP [Table 3]. Details for each round are provided below.

**Table 3:** Final list

Round3-No.	competency domain	definition of competency	explanation of competency
R3–1.	Cultural competence	can understand the diverse socio-economic status and cultural background of patients and provide care that takes into account diverse medical needs.	understand the diverse socio-economic status (social status, education level, lifestyle, occupation, income, insurance, etc.) and cultural background (race, religion, thought, beliefs, customs, etc.) of patients and provide care that takes into account the diverse medical needs that accompany the situation.
R3–2.	Care for people at a social disadvantage	can understand social determinants of health and health inequalities, and can work with multiple occupations to provide appropriate care to a wide variety of populations with inadequate medical care.	understand social determinants of health and health inequalities, and can work with multiple occupations to provide appropriate care to a wide variety of populations with inadequate medical care (social isolation, withdrawal, poor areas, low income, uninsured, homeless, race/ethnic minorities, immigrants, LGBT, HIV/AIDS patients, commercial sex workers, criminal history, etc.)
R3–3.	Family-oriented care	can consider diverse values ​​and relationships with the family, communicate effectively with the necessary stakeholders, and provide appropriate care for the patient.	share information about the patient’s medical condition and make important decisions by taking into consideration various values and relationships with family members (not limited to blood relatives, but including common-law relatives and close acquaintances, various household structures and residential situations, etc.) and by communicating effectively with necessary parties. By communicating effectively with the necessary parties, they are able to provide support for decision-making that is appropriate for the patient and appropriate care.
R3–4.	Adjustment of the scope of care	can flexibly adjust the scope of care they provide to meet the diverse needs and problems of patients, taking into account the characteristics of a wide variety of surrounding medical institutions.	GPs can flexibly adjust the scope of care they provide to meet the diverse needs and problems of patients in terms of expanding or contracting the scope of care based on the characteristics of the various surrounding medical institutions (segmented specialties, trends in treatment policies, access, etc.) while maintaining a complete picture of the patient.
R3–5.	Coordination of care with specialized medical institution	can grasp the characteristics of a wide variety of medical institutions with fragmented specialties, and make appropriate referrals to and coordinate care with specialized medical institutions according to the needs and circumstances of the patient.	GPs can grasp the characteristics of a wide variety of medical institutions with fragmented specialties, and make appropriate referrals and linkages to specialized medical institutions according to patients’ diverse needs and circumstances (medical conditions, underlying diseases, socioeconomic status, transportation).
R3–6.	Integration of fragmented medical care	can take responsibility for the integration of medical care for patients who are experiencing the negative effects of fragmentation of care.	can take responsibility for organizing medical visits and providing comprehensive care to patients who have multiple diseases and are suffering from the negative effects of fragmented care due to visits to multiple specialties, by building trusting relationships and collaborating with doctors in specialties and other professionals inside and outside the facility.
R3–7.	Coordination of care with multiple professions	can grasp the characteristics of a wide variety of care and welfare services and community social resources, and make appropriate referrals and collaborations in collaboration with multidisciplinary professionals according to the needs and circumstances of the patient.	can grasp the characteristics of a wide variety of care and welfare services (e.g., nursing homes, long-term care facilities, home nursing agencies) and community resources (e.g., civic activities, hobby groups), and can make appropriate referrals and collaborate with them according to the patient’s situation in collaboration with multidisciplinary professionals (e.g., care managers, nurses, community comprehensive support center staff).
R3–8.	Community Oriented Care -Health Promotion	can identify health issues that are characteristic of the region/community and effectively collaborate with a wide variety of stakeholders to address them.	can identify health issues that are characteristic of the region and community in which they practice, and work effectively with a wide variety of stakeholders, including the people concerned, surrounding residents, and multidisciplinary professionals, to address health issues through ongoing planning, implementation, and evaluation. For example, it is possible to address the health problems of specific groups, such as poor areas, areas with frequent ambulance use, elderly single households, foreign residents, night shift workers, and single-parent families.
R3–9.	Community Oriented Care -emergency care	can collaborate and tackle the issues of emergency medicine that are characteristic of each region at the field level of primary care.	can share issues with related parties (hospitals, clinics, ambulance crews, etc.) regarding emergency medical care in each medical area, cooperate from the field level of primary care, and implement some measures. For example, there are discussions on cases of tampering with emergency patients, efforts to ensure the continuity of patient information during emergency consultations on holidays and nights, and measures for patients who frequently undergo emergency consultations.
R3–10.	Details -Occupational health	can provide appropriate care for occupational health-related health problems that are common or characteristic of each practice area.	can provide appropriate care as an industrial physician or in collaboration with an industrial physician for health problems related to industrial hygiene that are common or characteristic in each medical area. For example, not only chemical and physical health disorders such as organic solvents, dust, noise, and vibration, but also psychosocial health disorders such as mental health and overwork, ergonomic health disorders such as VDT work and working attitude, or overseas workers. It is possible to flexibly respond to different needs depending on the medical treatment area, such as biological health problems like measures against infectious diseases.
R3–11.	Details-Infectious diseases	The GP can identify patients with suspected frequent infections in urban areas and take appropriate initial action.	GP can identify patients with symptoms and risk factors that should be suspected to be frequent infectious diseases in urban areas such as tuberculosis and sexually transmitted diseases such as HIV infection, and recall those diseases as a differential diagnosis, which is necessary. Appropriate initial measures such as conducting tests and coordinating/introducing with health centers and specialized medical institutions can be performed.
R3–12.	Details-Mental Health	can respond appropriately to mental health problems in patients of all ages and collaborate with psychiatrists and related agencies.	can respond appropriately to mental health problems in patients of all ages (especially children and young people with developmental disabilities, school refusal and elderly depression, delirium, or multi-generational drinking, smoking, drug addiction, etc.) It is possible to deal with various primary care levels and cooperate with psychiatry and related organizations.
R3–13.	Details-Dementia care	can carry out appropriate diagnosis and treatment for patients suspected of having dementia, and care management in collaboration with multiple occupations.	can appropriately cooperate with specialists in the diagnosis and drug treatment for patients suspected of having dementia, and can cooperate with multiple occupations (nurses, rehabilitation workers, care managers, caregivers, etc.) in care management to improve the quality of life, and also appropriately cooperate with administrative procedures as necessary (such as the written opinion of the attending physician for the use of long-term care insurance and the preparation of documents for the adult guardianship system).
R3–14.	Details-Behavioral transformation	can provide guidance using behavior modification theory to patients with lifestyle-related diseases.	can provide guidance to patients with lifestyle-related diseases using behavioral change theory based on various lifestyles.
R3–15.	Details-Palliative care	can provide appropriate decision support and palliative care to patients with cancer or non-cancerous diseases.	can continuously and appropriately support the decision-making of the patient, their family, or the surrogate decision-maker for patients in the treatment stage to the terminal stage of cancer or non-cancer disease. In addition, it is possible to provide appropriate palliative care at the place (home, admission facility, hospital, hospice, etc.) according to the patient’s wishes and circumstances while coordinating and coordinating with each person concerned.
R3–16.	Organization management	can work on the organizational management of clinics and hospitals based on the role of primary care in each region.	can work on appropriate organizational management of clinics and hospitals (improvement of patient convenience, improvement of quality of medical care provided, division of roles with surrounding medical institutions and network formation, etc.) based on the role of primary care in each region.
R3–17.	Lifelong learning	GPs can learn about common illnesses to maintain their ability to practice regardless of the frequency of treatment opportunities.	can intentionally learn to maintain competence in common diseases, including emergencies and chronic diseases, regardless of the frequency of opportunities to practice in a setting with good access to surrounding specialty care facilities.
R3–18.	Education	can provide opportunities to learn the characteristics and significance of urban primary care in student and internship education.	can provide opportunities to learn the characteristics and significance of urban primary care at various student and internship education opportunities in community medical training. For example, through clinical training in urban areas, it is possible to provide an opportunity to learn that primary care is necessary not only in non-urban areas and depopulated areas but also in urban areas.

#### Round 1 of the Delphi method

In the first round, we received responses from all 39 panelists. Of the initial list of 34 items, two items, “Item 30. Refugee psychiatric problems” and “Item 34. Traffic trauma,” were excluded because they had means 3.5 or less or standard deviations > 1. Based on the panelists’ opinions, the research team then reviewed the list and merged it as follows. Items 1–3 were identified as “cultural competence,” 4–6 as “urban underserved care,” 10–14 as “integration of care,” and 15,16 as “coordination of care with multiple professions”. In addition, the panelists’ revised opinions were used as the basis for the analysis. Based on these revised opinions, we re-edited the competency descriptions in three levels: “competency domain,” “definition of competency,” and “description of competency” [26]. Additionally, we reorganized six new competencies that were not on the initial list suggested by the panelists. As a result, we obtained a list of 20 items in the first round.

#### Round 2 of the Delphi method

In Round 2, 38 of 39 panelists (97.4%) responded to the survey. Including the new items suggested by the panelists in Round 1, there were no items on the list that met tra each item had a high level of agreement with a mean value of 4.0 or higher. The research team qualitatively analyzed and discussed the participants’ opinions on each competency, revised the descriptions of each competency, and integrated items 12 and 17, mental health, and 15 and 16, palliative care, resulting in a list of 18 items.

#### Round 3 of the Delphi method

In Round 3, 36 (92.3%) of the 39 panelists responded to the survey. In Round 3, all 18 items had a mean value of 4.0 or higher, and no excluded items with a standard deviation > 1 were found. Therefore, the survey was considered to have reached a consensus among all participants and was terminated in the third round. Finally, the opinions of the participants on each competency were qualitatively analyzed by two researchers using the thematic analysis method, and the wording of the list was partially revised. Since the list had already been agreed upon, no consolidation or deletion of the list was done. The result was a list of 18 items as competencies specifically needed by urban GP/FP [Table 3].

## Discussion

### Summary

This study aimed to identify a validated and comprehensive list of competencies specifically needed by urban GP/FP. Using a modified Delphi method, we developed an initial list through a preliminary literature review and conducted a total of three rounds of interactive refinement with 39 expert panelists. These resulted in an 18-items competency list guaranteeing a certain level of content validity in the research process.

### Strength of this study

There are two strengths of this study. First, it is a novel study examining a validated and comprehensive list of urban GP/FP competencies which have not been adequately investigated. While there have been several partial or empirical lists of competencies for urban GP/FP, none have explicitly described the literature review and comprehensive list generation through research methods. This study reflected the literature review and the diverse opinions of panelists from three rounds of the Delphi method to obtain a broad and detailed list of competencies for urban GP/FPs. Second, we have identified some new competencies not previously identified as urban GP/FP. The ability to coordinate and integrate various care resources fragmented due to overconcentration in urban areas and the ability to educate future generations about the significance of urban primary care are newly identified as important. This will be discussed below by comparing representative existing literature.

### Comparison with existing literature

We discuss a representative list of 18 competencies obtained. One known competency of urban GP/FP is “cultural competence” (R3–1). It is the ability to understand cultural diversity and provide appropriate care in clinical practice. It is known as an important competency in healthcare strategies to reduce inequalities due to cultural differences and provide quality care to all people [7, 27–31]. Another representative of the known competencies of urban GP/FP is “care for the socially disadvantaged” (R3–2.). This is the ability to provide appropriate care to the urban underserved communities, especially those who have difficulty accessing health care, such as the homeless, uninsured, poor, immigrants, and sexual minorities. This has been a growing concern with the recent accumulation of knowledge on the social determinants of health [32–34].

On the other hand, the four competencies of “coordination of care with specialized medical institutions” (R3–5.), “coordination of care with multiple professions” (R3–7.), “adjustment of scope of practice” (R3–4.), and “integration of fragmented medical care” (R3–6.) indicate the need for GP/FP to have the ability to “provide integrated care” in response to “fragmentation of care,” a side effect of specialization and overconcentration of care resources associated with urbanization [35, 36]. In particular, it refers to integrating care at the micro (clinical integration) and meso (professional and organizational) levels [37]. Although integrated care itself is not a new concept, it has not received as much attention as cultural competency and urban underserved care as a competency of urban GP/FP. This is a new competency that we were able to focus on in this study.

In addition, new competencies added based on the panelists’ opinions include palliative care (R3–15), organizational management (R2–16), lifelong learning (R2–17), and education (R2–18). Especially for education (R2–18), many panelists as urban GP/FP emphasized the importance of “providing opportunities to learn about the need and significance of primary care not only in rural but also in urban areas, regardless of future careers,” a distinctive competency not found in the existing literature.

### Limitations and challenges for further study

This study has several limitations and challenges for further research. One is the number of panelists. The number of panelists in this study was 39, possibly a relatively small number. However, since there is no strict standard for the number of panelists in the Delphi method [14], we set the number based on recent medical education studies [38, 39]. Also, it could be evaluated to a certain extent that this study finally obtained a high response rate of 36 out of 39 (92.3%). In the future, it is desirable to evaluate the validity of the list with more panelists, considering the balance between the number of participants and the dropout rate. Another limitation is the quality of the panelists. Of the GP/FP panelists, 6 out of 26 selected the female gender option, and none selected the “neither” option. As of 2019, when the survey was conducted, the percentage of female physicians by gender in Japan was 21.9%, which is not significantly different from the percentage in this study, but very low compared to the OECD average. This gender gap is one of the issues in Japan that needs to be improved. Since this gender imbalance may have affected the selection of competencies in this study, further research with a more balanced gender balance is desirable. There were only three patient representatives as panelists, and the opinions of other professionals were not reflected. It is hoped that a more diverse list of stakeholders will be included in the future [19]. In addition, this study was conducted in a metropolitan area in Japan, and there was a limit to the possibility of transferring the results to other countries and cities with different demographics and healthcare systems. However, by using the results of this study as a comparison, it will be easier to reevaluate and compare the validity of the list in other countries and cities. There is also the methodological limitation of the Delphi, which is the selection of specific panelists at a specific time. Since the responses may be influenced by the situation and interests of the panelists at the time of the survey, for example, if a situation such as a disaster or infectious disease outbreak in an urban area occurs, consideration should be given to the possibility that these may be listed as candidates for competency or given a strong weighting. It is necessary to periodically revise the list among the stakeholders involved in the field implementation. In fact, this study is both limited and valuable in that the data were collected and analyzed before the outbreak of COVID-19 infection, which had a significant impact on the GP/FP role [40].

### Prospects for further practice and research

In order to use the list of competencies from this study to inform the clinical practice of urban GP/FP, it is necessary to develop an educational curriculum and content for urban primary care that incorporates specific educational strategies and assessment methods. Action research is also desired to evaluate whether educational practices based on these curricula lead to the training of high-quality urban GP/FP. Finally, it is necessary to evaluate whether developing quality urban GP/FP can contribute to improving health outcomes for people living in cities [41].

## Conclusion

The competency list obtained in this study covers a wide range of competency areas required as a GP/FP and is more developed in defining and describing competencies, reflecting the characteristics of urban areas compared to general GP/FP competencies. Particularly, in addition to the known competencies of an urban GP/FP, such as cultural competence and caring for socially disadvantaged populations in urban areas, we newly identified the importance of the ability to coordinate and integrate various care resources that are highly fragmented in urban areas.

With global urbanization, training GP/FP who can effectively practice urban primary care will become an important issue in many countries. The comprehensive list of competencies presented in this study can serve as an indicator for policymakers and education program managers to consider what kind of urban GP/FP should be trained. For GP/FP who understand their patients’ backgrounds and practice patient-centered medicine, the list of competencies presented in this study may provide them with a bird’s-eye view of their role in urban primary care, a way to reflect on their clinical practice, and a guideline for providing effective primary care. This will ultimately lead to a healthier life for all people living in cities.

## Supplementary Information

**Additional file 1.**

## Data Availability

The datasets used and analyzed during the current study are available from the corresponding author on reasonable request.

## Abbreviations

GP: general practitioner
FP: family physician
PGY: post-graduate year
