# Supplementary material for: Competency lists for urban general practitioners/family physicians using the modified Delphi method
Source: BMC Prim Care. 2023 Jan 19;24:21. doi: 10.1186/s12875-023-01984-z (PMC9849100; doi:10.1186/s12875-023-01984-z)
Supplement: Supplementary file 1 — Additional file 1. [file 12875_2023_1984_MOESM1_ESM.docx]

**Additional file 1. The formula of literature search**


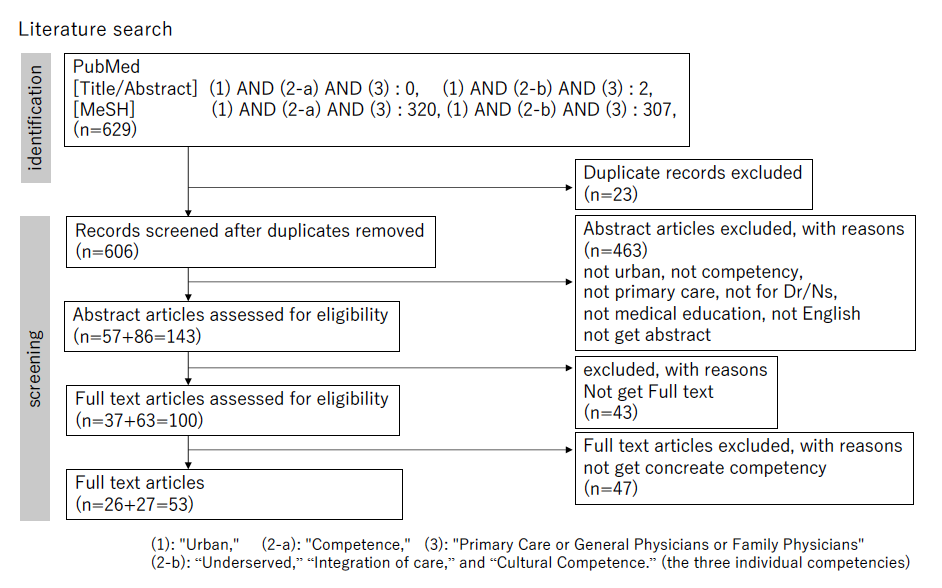


**Additional file 2. Descriptive statistics and list editing process for each round in the modified Delphi method**

| Round1-No. | Competency domain | Result:  Mean value,  (Standard deviation),  Rank by mean | R2-No. | Competency domain | Result:  Mean value,  (SD),  Rank by mean | R3-No. | Competency domain | Result:  Mean value,  (SD),  Rank by mean |
| --- | --- | --- | --- | --- | --- | --- | --- | --- |
| R1-1. | Cultural competence (CC) | 4.44 (0.709) 11 | R2-1. | Cultural competence | 4.55 (0.497) 5 | R3-1. | Cultural competence | 4.5 (0.645) 11 |
| R1-2. | Cultural competence | 4.58 (0.494) 5 |  |  |  |  |  |  |
| R1-3. | Cultural competence | 4.59 (0.542) 1 |  |  |  |  |  |  |
| R1-4. | Urban underserved care (UUC) | 4.31(0.685) 15 | R2-2. | SDH, UUC, CC | 4.68 (0.518) 2 | R3-2. | Care for people at a social disadvantage | 4.58(0.546) 7 |
| R1-5. | UUC | 4.37(0.741) 12 |  |  |  |  |  |  |
| R1-6. | UUC | 3.63(0.840) 32 |  |  |  |  |  |  |
| R1-7. | Family-oriented care | 4.28(0.749) 16 | R2-3. | Family-oriented care | 4.55(0.714) 6 | R3-3. | Family-oriented care | 4.39 (0.792) 15 |
| R1-8. | Comprehensive care | 4.59 (0.741) 1 | R2-4. | Comprehensive care | 4.5 (0.59) 7 | R3-4. | Adjustment of the scope of care | 4.61 (0.636) 4 |
| R1-9. | Coordination of care | 4.49 (0.78) 6 | R2-5. | Coordination of care | 4.61(0.489) 3 | R3-5. | Coordination of care with specialized medical institution | 4.67 (0.471) 2 |
| R1-10. | Integrated care | 4.59 (0.669) 1 | R2-6. | Integrated care | 4.74(0.497) 1 | R3-6. | Integration of fragmented medical care | 4.78 (0.416) 1 |
| R1-11. | Integrated care | 4.47 (0.752) 8 |  |  |  |  |  |  |
| R1-12. | Integrated care−mental health | 4.21(0.853) 18 |  |  |  |  |  |  |
| R1-13. | Integrated care−pediatrics | 4.1 (0.81) 22 |  |  |  |  |  |  |
| R1-14. | Integrated care−HIV | 3.69 (0.91) 30 |  |  |  |  |  |  |
| R1-15. | Coordination of care | 4.49 (0.78) 6 | R2-7. | Coordination of care、multidisciplinary cooperation | 4.58 (0.591) 4 | R3-7. | Coordination of care with multiple professions | 4.58 (0.682) 6 |
| R1-16. | Multidisciplinary cooperation | 4.59 (0.669) 1 |  |  |  |  |  |  |
| R1-17. | Community Oriented Care | 4.46 (0.711) 9 | R2-8. | Community Oriented Care | 4.16 (0.708) 16 | R3-8. | Community Oriented Care -Health Promotion | 4.61 (0.591) 5 |
| R1-18. | Community Oriented Care - emergency care | 4.33 (0.613) 14 | R2-9. | Community Oriented Care - emergency care | 4.21 (0.832) 15 | R3-9. | Community Oriented emergency care | 4.44 (0.685) 13 |
| R1-19. | Biomedical problems – Occupational health | 4.08 (0.888) 23 | R2-10. | Occupational health | 3.95 (0.944) 20 | R3-10. | Details  − Occupational health | 4.17 (0.764) 18 |
| R1-20. | Biomedical problems – Infectious diseases | 4.18 (0.712) 20 | R2-11. | Biomedical problems – Infectious diseases | 4.42 (0.634) 10 | R3-11. | Details-Infectious diseases | 4.53 (0.499) 9 |
| R1-21. | – Infectious diseases | 3.69 (0.821) 30 |  |  |  |  |  |  |
| R1-22. | – Infectious diseases | 4 (0.784) 24 |  |  |  |  |  |  |
| R1-23. | – Infectious diseases | 3.97 (0.862) 25 |  |  |  |  |  |  |
| R1-24. | Mental health-adolescence | 4.13 (0.822) 21 | R2-12. | Mental health | 4.32 (0.765) 11 | R3-12. | Details-Mental Health | 4.53 (0.687)10 |
| R1-25. | − Pediatric care | 3.9 (0.778) 27 |  |  |  |  |  |  |
| R1-26. | − Pediatric care | 3.9 (0.841) 27 |  |  |  |  |  |  |
| R1-27. | − Elderly care | 4.36(0.832) 13 |  |  |  |  |  |  |
| R1-28. | − Elderly care | 3.82(0.873) 24 |  |  |  |  |  |  |
| R1-29. | − Cultural competence | 3.95 (0.749) 26 |  |  |  |  |  |  |
| R1-30. | − Cultural competence | 3.38 (0.923) 33 |  |  |  |  |  |  |
| R1-31. | – Elderly care−Dementia | 4.46 (0.812) 9 | R2-13. | Elderly care−Dementia | 4.47 (0.752) 8 | R3-13. | Details-Dementia care | 4.42 (0.722) 14 |
| R1-32. | – Elderly care−Dementia | 4.23 (0.799) 17 |  |  |  |  |  |  |
| R1-33. | Behavioral medicine | 4.21 (0.853) 18 | R2-14. | Behavioral change | 4.05 (0.944) 18 | R3-14. | Details-Behavioral change | 4.36(0.787) 16 |
| R1-34. | Biomedical problems-surgery | 3.13 (0.911) 34 |  |  |  |  |  |  |
|  |  |  |  | <New competencies> |  |  |  |  |
|  |  |  | R2-15. | End of Life care, Palliative Care | 4.26 (0.784) 13 | R3-15. | Details-Palliative care | 4.47(0.799) 12 |
|  |  |  | R2-16. | Communication – end of life | 4.32 (0.798) 12 |  |  |  |
|  |  |  | R2-17. | Psychiatric problems − Addiction | 4.08 (0.703) 17 |  |  |  |
|  |  |  | R2-18. | Organization management | 4.24 (0.705) 14 | R3-16. | Organization management | 4.31(0.907) 17 |
|  |  |  | R2-19. | Lifelong learning | 4 (0.889) 19 | R3-17. | Lifelong learning | 4.53 (0.499) 8 |
|  |  |  | R2-20. | Education | 4.47 (0.595) 9 | R3-18. | Education | 4.64 (0.535) 3 |
